# Upregulation of Tribbles decreases body weight and increases sleep duration

**DOI:** 10.1242/dmm.049942

**Published:** 2023-04-21

**Authors:** Rebeka Popovic, Yizhou Yu, Nuno Santos Leal, Giorgio Fedele, Samantha H. Y. Loh, L. Miguel Martins

**Affiliations:** MRC Toxicology Unit, University of Cambridge, Gleeson Building, Tennis Court Road, Cambridge CB2 1QR, UK

**Keywords:** *Drosophila*, Tribbles, Lipid metabolism, Sleep, Fat body, Lifespan

## Abstract

Eukaryotic Tribbles proteins are pseudoenzymes that regulate multiple aspects of intracellular signalling. Both *Drosophila melanogaster* and mammalian members of this family of pseudokinases act as negative regulators of insulin signalling. Mammalian tribbles pseudokinase (TRIB) genes have also been linked to insulin resistance and type 2 diabetes mellitus. Type 2 diabetes mellitus is associated with increased body weight, sleep problems and increased long-term mortality. Here, we investigated how manipulating the expression of Tribbles impacts body weight, sleep and mortality. We showed that the overexpression of *Drosophila tribbles* (*trbl*) in the fly fat body reduces both body weight and lifespan in adult flies without affecting food intake. Furthermore, it decreases the levels of *Drosophila* insulin-like peptide 2 (DILP2; ILP2) and increases night-time sleep. The three genes encoding TRIBs of mammals, *TRIB1*, *TRIB2* and *TRIB3*, show both common and unique features. As the three human TRIB genes share features with *Drosophila trbl*, we further explored the links between TRIB genetic variants and both body weight and sleep in the human population. We identified associations between the polymorphisms and expression levels of the pseudokinases and markers of body weight and sleep duration. We conclude that Tribbles pseudokinases are involved in the control of body weight, lifespan and sleep.

## Teaser

**Summary:** *In vivo* analysis in *Drosophila*, combined with *in silico* analysis of human genetic variants, reveals that Tribbles, which is involved with cell growth and division, metabolism, inflammation and cancer, also controls body weight and sleep.

## INTRODUCTION

Obesity is currently one of the most significant global health concerns. Obesity, characterised by increased accumulation of lipids in adipose tissue, leads to insulin resistance and metabolic abnormalities associated with the development of type 2 diabetes mellitus (T2DM) (Eckel et al., 2011).

Diabetes is a leading cause of death worldwide; in 2017, over 6% of the global population had T2DM, a disorder in which the body becomes less responsive to insulin, a key regulator of anabolic metabolism (Khan et al., 2020). In contrast to type 1 diabetes, an autoimmune condition that destroys insulin-producing β cells in the pancreas, T2DM is linked to lifestyle factors, such as a poor diet and lack of physical activity, and leads to poor sleep and an increased risk of mortality in adults (Lee et al., 2017; Lou et al., 2012; Wang et al., 2020). Alterations in the insulin receptor signalling (IRS) pathway lead to insulin resistance and T2DM. Insulin acts on its target cells by activating protein kinase B (Akt) (Cross et al., 1995).

*Drosophila melanogaster* is used as a model to study fat metabolism and diabetes (reviewed by Musselman and Kühnlein, 2018). Similar to humans, *Drosophila* individuals become obese when raised on a high-fat diet (HFD) and develop a diabetic phenotype (Musselman et al., 2011).

Tribbles pseudokinases are a family of adaptor proteins that modulate the activity of transcription factors and kinases. They are associated with several human diseases, including diabetes, lipid disorders and sleep disturbances (reviewed by Kiss-Toth, 2011). Three distinct tribbles pseudokinase (TRIB) genes have evolved in mammals, tribbles pseudokinases 1, 2 and 3 (*TRIB1*, *TRIB2* and *TRIB3*), which exhibit both unique and shared features, whereas *Drosophila* has only a single gene in this family, *tribbles* (*trbl*) (Eyers et al., 2017). Human TRIB3 has been shown to regulate insulin signalling by acting as an inhibitor of Akt (Du et al., 2003; Koo et al., 2004), and the presence of a single-nucleotide polymorphism (SNP) in *TRIB3* is reported to be a risk factor for T2DM (Prudente et al., 2009). *Drosophila* Trbl, like TRIB3, can also act as an Akt inhibitor by preventing the phosphorylation of Akt at the conserved phospho-threonine 308 site (Das et al., 2014). In *Drosophila*, overexpressing *trbl* in the fat body mimics metabolic defects induced by a HFD, and its downregulation in the fat body alleviates the metabolic defects associated with a HFD, indicating that Trbl mediates insulin resistance caused by this diet in flies (Hong et al., 2016). The expression of Trbl in *Drosophila* is under the control of the transforming growth factor β (TGFβ) signalling pathway (Hong et al., 2016).

Given the role of Trbl in the regulation of pathways linked to T2DM, we investigated whether manipulating its expression impacted insulin signalling, body weight, sleep and lifespan. Here, we combine an *in vivo* analysis, in which we manipulated the expression levels of *Drosophila* Trbl, with an *in silico* analysis of variants of human *TRIB1*, *TRIB2* and *TRIB3*. First, we show that, in adult flies, increasing the expression of *trbl* results in decreases in fly body weight, lifespan and systemic insulin levels and an increased sleep duration. Second, based on the premise that the three human TRIBs share common features (Eyers et al., 2017), we show through causal inference (Mendelian randomisation) that variants across the three kinases are linked to an inverse relationship between markers of obesity and sleep duration.

## RESULTS

### Ubiquitous expression of Trbl decreases lifespan and motor function

**Fig. 1.**
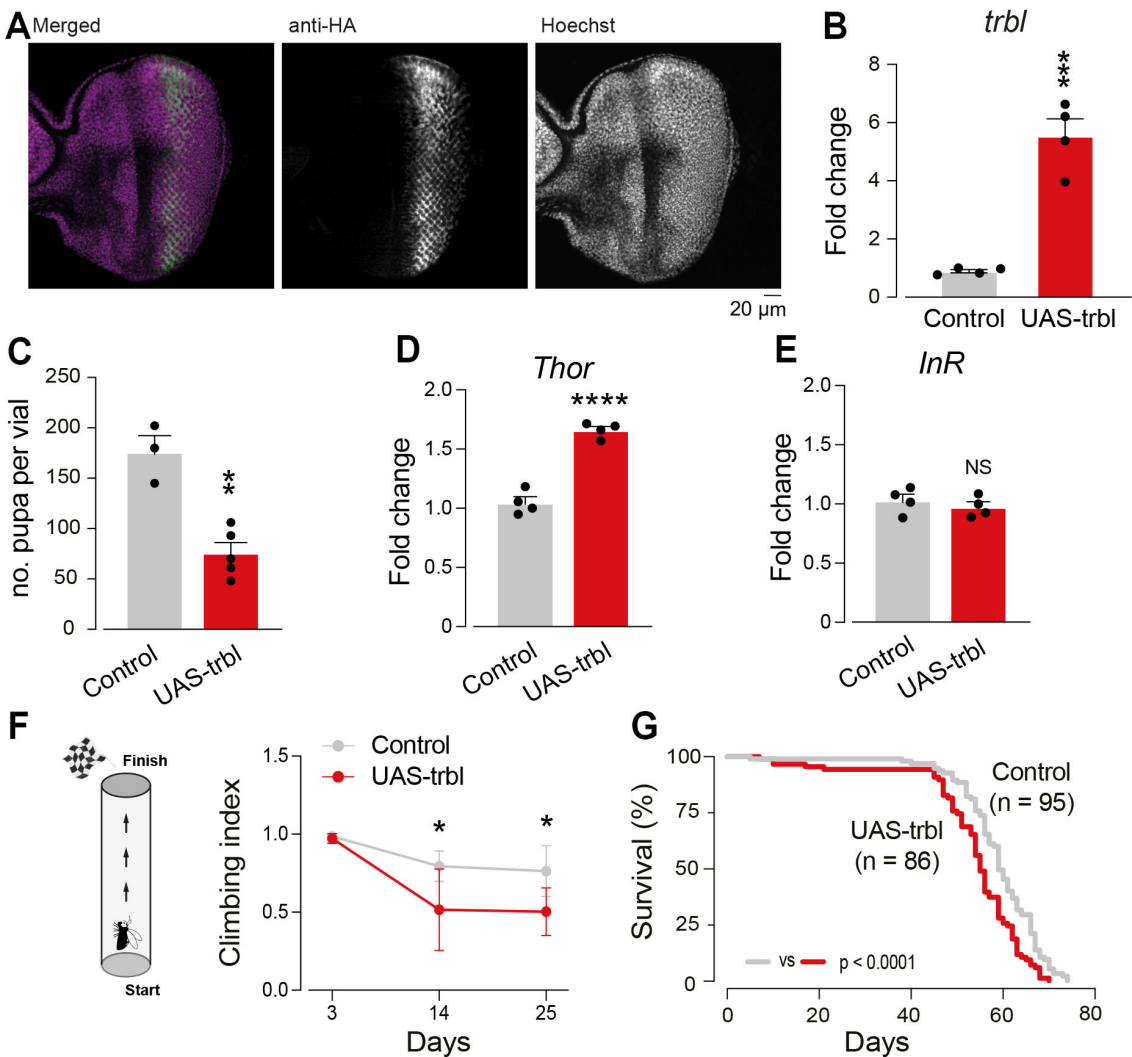
**Increased expression of *tribbles* (*trbl*) causes motor impairment and decreases survival.** (A) Analysis of Trbl expression in larval eye discs. Third instar eye imaginal discs of UAS-*trbl*-HA flies driven by *gmr*-Gal4, immunostained for HA (green) and Hoechst (purple). (B) Ubiquitous expression of the UAS-*trbl*-HA leads to increased *trbl* transcript levels. mRNA levels were measured by real-time qPCR (mean+s.e.m.; ***P<0.001; unpaired *t*-test; *n*=4 biological replicates). (C) Ubiquitous expression of *trbl* causes a delay in development, with reduced numbers of developed pupae (mean+s.e.m.; **P<0.01; unpaired *t*-test). (D,E) Ubiquitous expression of *trbl* leads to transcriptional upregulation of *Thor* (D) (mean+s.e.m.; ****P<0.0001; unpaired *t*-test; *n*=4 biological replicates), but not *InR* (E) (mean+s.e.m.; NS, not significant; unpaired *t*-test; *n*=4 biological replicates). Transcripts were measured in 14-day-old adult males. (F) Ubiquitous expression of *trbl* decreases motor performance. Adult flies were tested using a standard climbing assay (mean±s.e.m.; *P<0.05; two-way ANOVA with Sidak multiple comparisons test; *n*≥5 biological replicates). (G) Ubiquitous *trbl* expression reduces lifespan (log-rank test). Genotypes: in A, *w; gmrGal4/UAStrbl-HA;+*; in B-G, *w; +; daGal4/+* (control), *w; UAStrbl-HA/+; daGal4/+* (UAS-trbl).

*Drosophila* is a useful organism for modelling obesity and metabolic diseases in humans. The conservation of the IRS pathway between flies and humans allows examination of the links between nutrient homeostasis, energy metabolism and IRS (Koyama et al., 2013; reviewed by Musselman and Kühnlein, 2018). To study further the potential role of Trbl in controlling metabolism, we examined the effects of its ubiquitous overexpression, using the *daughterless* (*da*) Gal4 driver in flies. We first confirmed the successful overexpression of *trbl* by measuring its protein and mRNA levels (Fig. 1A,B). Trbl has been shown to delay development and reduce cell size (Das et al., 2014); therefore, we examined the developmental consequences of overexpression of Trbl. We observed that overexpression of Trbl caused a decrease in the number of pupae at day 10 after egg laying (AEL), confirming that its expression compromises development (Fig. 1C).

Trbl has been reported to act as a negative regulator of insulin signalling by inhibiting Akt (Das et al., 2014), a protein kinase that phosphorylates and inhibits the transcription factor FOXO (Biggs et al., 1999). FOXO controls the transcription of *Thor* (also known as *4EBP*), and of Insulin-like receptor (InR) itself in a transcriptionally induced feedback control mechanism (Puig et al., 2003). Overexpression of Trbl has been shown to increase the expression of both Thor and InR in the fat body of flies (Hong et al., 2016). We therefore analysed the levels of the *Thor* and *InR* transcripts in adult flies and confirmed that overexpression of Trbl led to an increase in the expression of *Thor*, but not *InR* (Fig. 1D,E). Finally, we addressed the effects of the ubiquitous expression of Trbl on motor function and ageing. We found that Trbl expression caused an age-dependent loss of motor function (Fig. 1F) and a reduction in lifespan (Fig. 1G). In summary, our results show that the ubiquitous overexpression of this pseudokinase has detrimental effects on motor function and survival.

### Fat body expression of Trbl decreases body weight and increases resistance to starvation

**Fig. 2.**
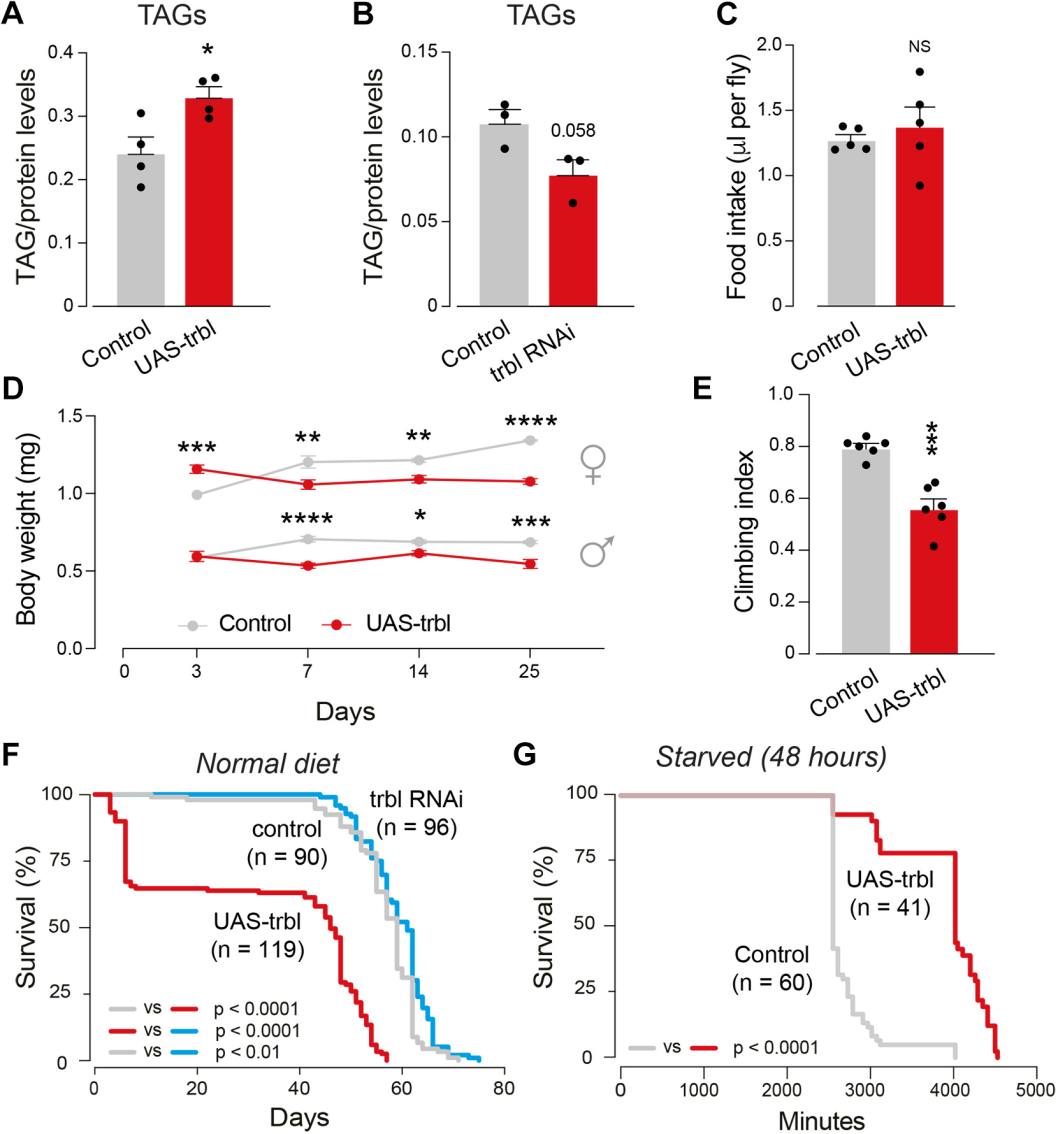
**Expression of *trbl* in the fat body decreases adult body weight and lifespan.** (A,B) *ppl*-Gal4-driven expression of *trbl* increases triglyceride (TAG) levels (A; mean+s.e.m.; *P<0.05; unpaired *t*-test), whereas *trbl* silencing decreases TAG levels (B; mean+s.e.m.; unpaired *t*-test). (C) Adult flies expressing *trbl* in the fat body show normal food intake (mean+s.e.m.; NS, not significant; unpaired *t*-test). (D) Expression of *trbl* in the fat body reduces body weight (mean±s.e.m.; *P<0.05, **P<0.01, ***P<0.001, ****P<0.0001; two-way ANOVA with Sidak multiple comparisons tests; *n*=3-5 biological replicates). (E) Fat body-directed *trbl* expression reduces motor performance. Flies were tested using a standard climbing assay (mean+s.e.m.; ***P<0.001; unpaired *t*-test). (F) *ppl*-Gal4 expression of *trbl* (red) in fully fed flies reduces whereas silencing *trbl* (blue) increases lifespan (log-rank test). (G) Expression of *trbl* in the fat body increases survival of stressed (starved) flies (log-rank test). All analyses (A-E) were conducted with 14-day-old male flies, unless otherwise specified. Genotypes: *w; pplGal4/+; +* (control), *w; pplGal4/UAStrbl-HA; +* (UAS-trbl), *w; pplGal4/UAS trblRNAi; +* (trbl RNAi).

Energy storage in flies involves the formation of triacylglycerols (TAGs), which are the main form of stored lipids in flies. TAGs are stored in the *Drosophila* fat body, an organ equivalent to the liver and adipose tissue in humans (Ugur et al., 2016). TAGs form lipid droplets in the fat body that can be mobilised as fuel to meet energy demands (reviewed by Zheng et al., 2016). Overexpression of Trbl in the fat body induces metabolic alterations characterised by an increase in TAGs (Hong et al., 2016). To define further the consequences of these metabolic alterations, we used the *pumpless* (*ppl*) Gal4 fat body driver and confirmed that the overexpression of Trbl using this driver increased the levels of TAGs (Fig. 2A). Conversely, silencing the expression of Trbl by RNA interference (RNAi) decreased the overall levels of TAGs (Fig. 2B). TAG levels can be controlled by nutrient intake (reviewed by Eickelberg et al., 2022). To determine whether the increase in TAGs observed upon overexpression of Trbl in the fat body was linked to increased nutrient intake, we next measured the food intake of adult flies. However, we found no differences in food consumption in flies in which Trbl was overexpressed using the *ppl* driver (Fig. 2C). Next, to determine the relationship between TAG accumulation and weight, we measured the body weight of adult flies during their lifespan. The results showed that fat body expression of Trbl led to a significant reduction in body weight (Fig. 2D). We also measured the motor activity and lifespan of adult flies and found that fat body expression of Trbl was sufficient to cause motor impairment (Fig. 2E) and decrease lifespan (Fig. 2F). Conversely, the RNAi-mediated downregulation of Trbl resulted in a small increase in lifespan (Fig. 2F). Stored lipids act as a source of energy when calorie intake is limited. Therefore, we next tested whether the TAG accumulation observed in flies expressing Trbl in the fat body improved their resistance to starvation-mediated stress. We found that fat body expression of Trbl resulted in increased resistance to starvation, as the flies lived longer (Fig. 2G). We conclude that the TAG accumulation caused by the fat body expression of Trbl results in leaner flies with a decreased lifespan and increased resistance to starvation.

### Expression of Trbl in the fat body leads to systemic repression of insulin signalling and increases night-time sleep

**Fig. 3.**
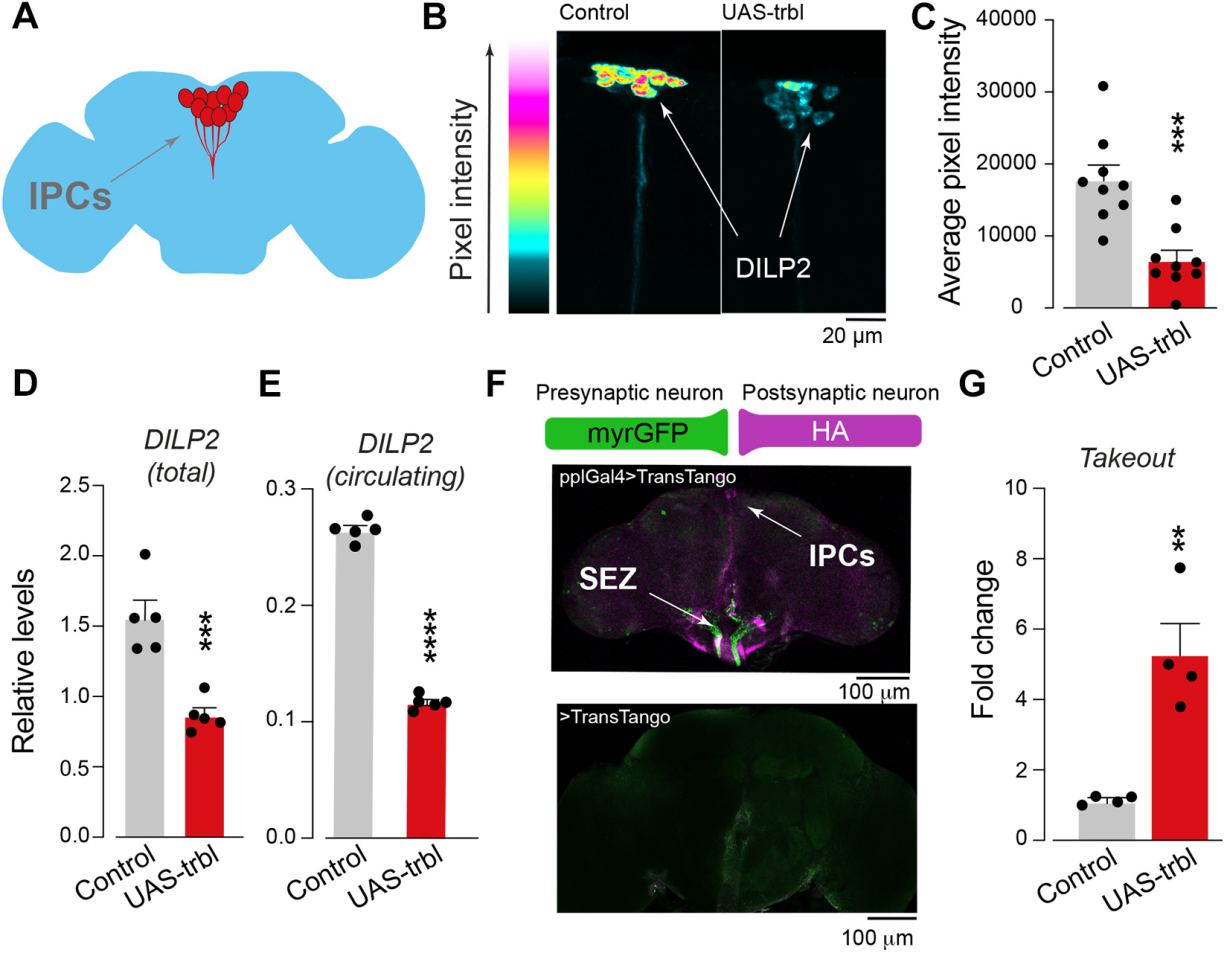
**Expression of *trbl* in the fat body increases markers of starvation and decreases DILP2 levels.** (A) Schematic representation of the anatomical location of the insulin-producing cells (IPCs, red) in the adult fly brain. Note that the IPCs are not to scale. (B,C) A reduced level of DILP2 was detected in IPCs of flies expressing *trbl* in the fat body. Representative images (B), with intensity levels shown as a five-tone heat map, and quantification (C) of the DILP2 signal intensity in IPCs (mean+s.e.m.; ***P<0.001; unpaired *t*-test, *n*=9). (D,E) Fat body-directed expression of *trbl* reduces total (D) and circulating (E) DILP2 levels. Total levels were measured from whole flies, and circulating levels were obtained from headless flies. (mean+s.e.m.; ***P<0.001, ****P<0.0001; unpaired *t*-test; *n*=5). (F) The brains of flies bearing the *trans*-Tango components, driving myrGFP expression under the control of *ppl*-Gal4 (green) exhibit mtdTomato::3xHA expression in postsynaptic IPCs (purple). Bottom panel shows a control brain carrying the *trans*-Tango components without the Gal4 driver. (G) *trbl* expression under the control of *ppl*-Gal4 leads to transcriptional upregulation of *takeout* (*to*), a marker of starvation (mean+s.e.m.; **P<0.01; unpaired *t*-test; *n*=4 biological replicates). Genotypes: in B-E, *w;pplGal4;Ilp2/+* (control), *w; pplGal4/UAStrbl-HA;Ilp2/+* (UAS-trbl); in F, *yw, UAS-myrGFP.QUAS-mtdTomato-3xHA; pplGal4/trans-Tango; +* (pplGal4>TransTango), *yw, UAS-myrGFP.QUAS-mtdTomato-3xHA; +/trans-Tango; +* (>TransTango); in G, *w; pplGal4;+* (control), *w; pplGal4/UAStrbl-HA;+* (UAS-trbl).

Trbl acts as an inhibitor of insulin signalling by blocking Akt, a downstream effector of this hormone (Hong et al., 2016). The fruit fly exhibits eight different insulin-like peptides (DILPs), four of which (DILP1, DILP2, DILP3 and DILP5) act similarly to human insulin. DILPs are produced by neuronal insulin-producing cells (IPCs) in the fly brain (Fig. 3A) that act like mammalian pancreatic β cells (Brogiolo et al., 2001). Given that Trbl overexpression in the fat body led to the accumulation of TAGs and leaner flies, we next examined whether insulin levels were altered by fat body expression of Trbl.

Confocal analysis of the fly brains showed that flies expressing Trbl in the fat body presented decreased levels of DILP2 in their IPCs (Fig. 3B,C). Next, we measured both total and circulating levels of DILP2 in adult flies and observed an overall reduction in these levels in flies expressing Trbl in the fat body (Fig. 3D,E).

To rule out the possibility that the downregulation of DILP2 observed in IPCs is a consequence of leaky expression of the *ppl*-Gal4 driver in that neuronal population, we coupled this driver to *trans*-Tango, a technique for trans-synaptic circuit tracing (Talay et al., 2017). Presynaptic expression driven by *ppl*-Gal4 was mostly observed in the subesophageal zone (SEZ), a primary centre for processing mechanosensory and gustatory inputs (Kendroud et al., 2018), and in the optic lobes, but not in IPCs. This expression pattern is not surprising, given that the *ppl* transcript is present in sensory neurons in the adult brain (Leader et al., 2018). However, although a postsynaptic signal was detected in the SEZ, we also observed positive staining in IPCs (Fig. 3F), suggesting a direct connection between *ppl-*expressing cells and insulin centres. Together, these results suggest that overexpression of Trbl in *ppl*-positive cells leads to systemic repression of insulin signalling. At present, it is still unknown whether this effect is mediated by fat body cells (via a humoral response) or through direct communication with neurons in the SEZ.

The secretion of DILPs by IPCs is decreased by starvation or low nutrient levels (Ahmad et al., 2019). Takeout is a *Drosophila* hormone controlled by the circadian clock that is induced upon starvation and is expressed in the fat body of flies. *Drosophila takeout* mutants have locomotor defects and die rapidly in response to starvation (Sarov-Blat et al., 2000). We previously observed an increase in Takeout levels and expression in flies with high levels of TAGs and lower levels of insulin (Fedele et al., 2022). Therefore, we next tested whether the overexpression of Trbl in the fat body was linked to an increase in this starvation marker. We measured the transcript levels of *Takeout* in flies in which Trbl was overexpressed using the *ppl* driver and found that they were significantly upregulated (Fig. 3G).

**Fig. 4.**
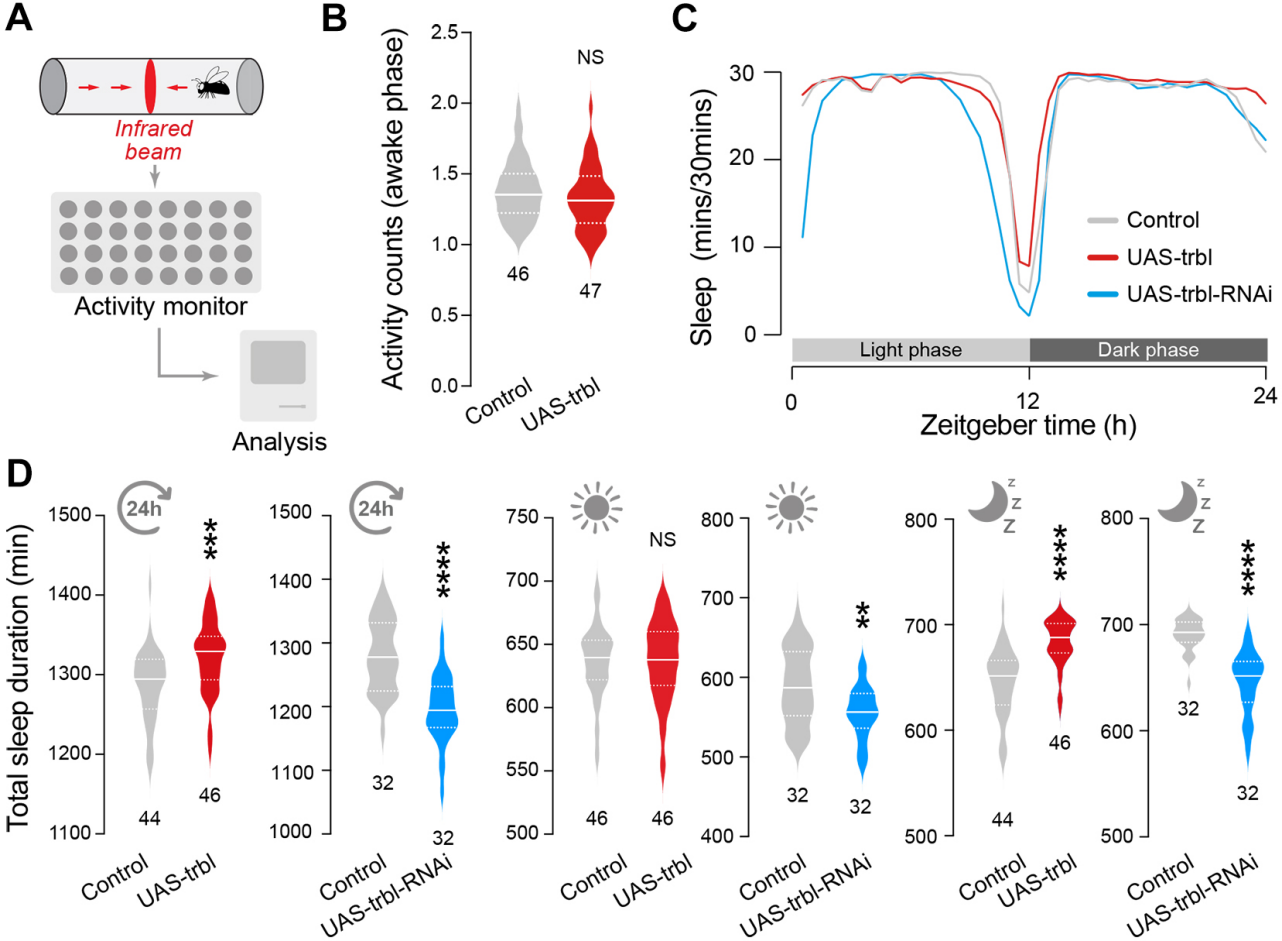
**Sleep is regulated by *trbl* levels in the fat body.** (A) Schematic representation of the *Drosophila* TriKinetics activity monitoring system. (B) Expression of *trbl* does not alter the activity of flies while they are awake (NS, not significant; unpaired *t*-test). (C) Analysis of circadian patterns of flies upon upregulation or downregulation of *trbl*. (D) *trbl* levels in the fat body regulate night-time sleep. Effects of *trbl* expression or RNAi on total sleep duration during 24 h (left; ***P<0.001, ****P<0.0001; unpaired *t*-test), during the day (middle; NS, not significant; **P<0.01; unpaired *t*-test) and during the night (right; ****P<0.0001; Mann–Whitney test) are shown. The analysis was carried out in 14-day-old male flies. Genotypes: in B-D, *w; pplGal4/+; +* (control), *w; pplGal4/UAStrbl-HA; +* (UAS-trbl), *w; pplGal4/UAS trblRNAi; +* (UAS-trbl-RNAi).

Next, we determined whether the observed increase in *takeout* expression caused by Trbl expression mediated by the *ppl* driver was linked to alterations in circadian patterns. We monitored the locomotor activity of adult flies under a standard light/dark cycle for 7 days using the *Drosophila* TriKinetics activity monitoring system (Fig. 4A). First, because we observed that Trbl expression in the fat body resulted in motor impairment (Fig. 2E), we assessed the awake activity of flies expressing Trbl using the fat body driver. There were no significant differences between these flies and the controls (Fig. 4B). Next, we analysed the sleep patterns of flies in which the *Trbl* transcript was either expressed or suppressed (Fig. 4C,D). We found that the expression of Trbl in the fat body increases sleep duration during the dark phase, but silencing Trbl in the fat body reduces sleep duration during both the light and dark phases (Fig. 4D).

We conclude that the expression of this pseudokinase causes a starvation-like phenotype characterised by a decrease in insulin levels, upregulation of a starvation-associated hormone and increased sleep duration.

### Genetic variations in the human TRIB family are linked to an inverse correlation between sleep and obesity

**Fig. 5.**
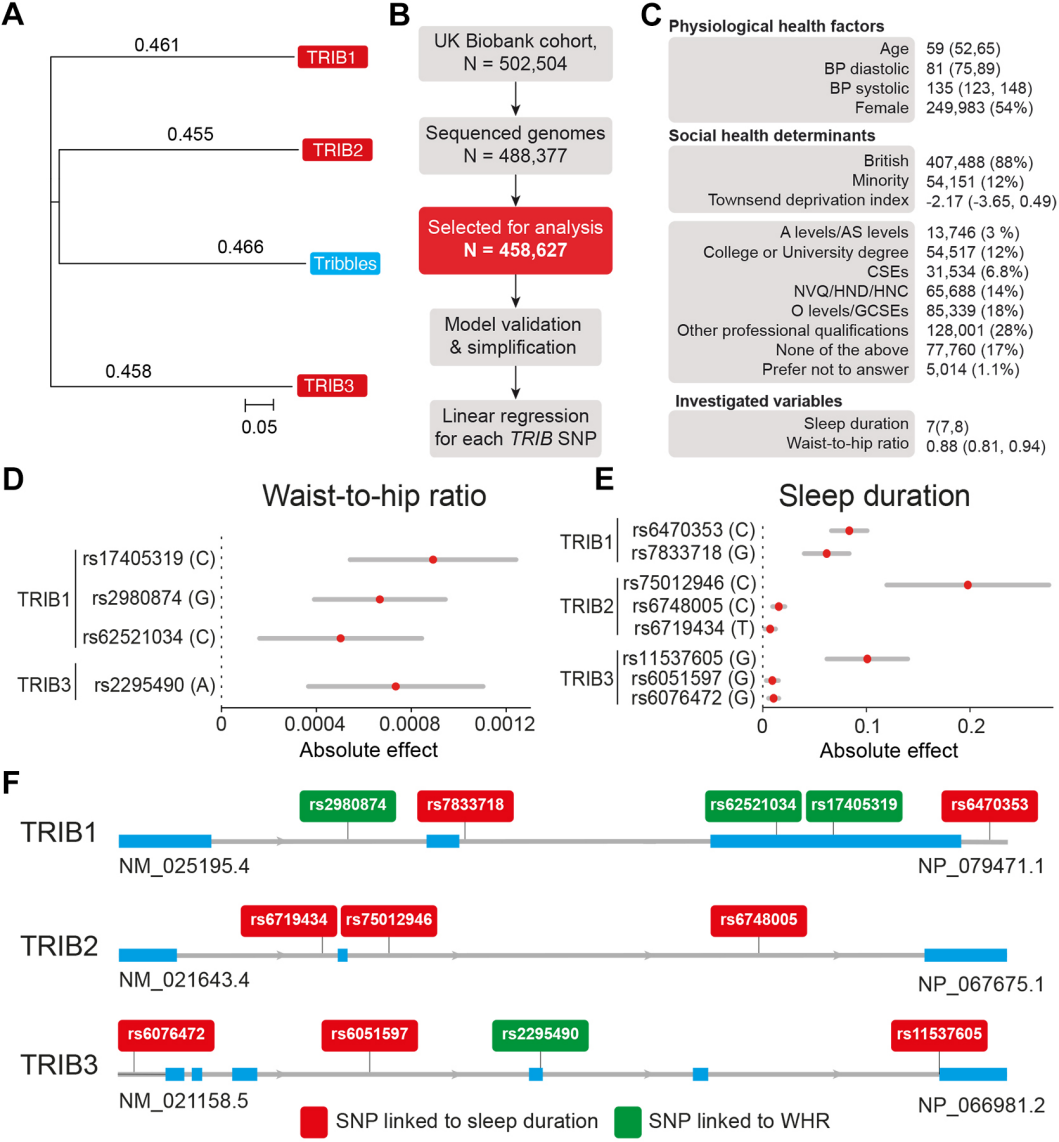
**Polymorphisms in human tribbles pseudokinase genes (*TRIB1-3*) are associated with alterations in sleep and obesity in the UK Biobank cohort.** (A) The *Drosophila tribbles* gene shows highest conservation with human *TRIB2*. Phylogenetic analysis between *Drosophila tribbles* (blue) and human *TRIB1-3* (red) was performed using the Neighbour Joining method. The scale corresponds to the distance between proteins in phylogenetic units, where a value of 0.05 corresponds to a difference of 5% between two sequences. (B) Workflow for the analysis of the participants of the UK Biobank cohort. After excluding participants with incomplete data, a subset of 458,627 individuals containing genomic and phenotypic data were selected for further analysis. (C) Descriptive statistics of the participants analysed in this study. Either the number of participants in each category and their percentage with respect to the total cohort, or the median and interquartile ranges are shown. (D,E) Absolute effect size of the significant SNPs in human TRIB genes associated with obesity (D) or sleep (E), following FDR correction using the Benjamini–Hochberg method. The red circles correspond to the absolute effect size of the SNP and the grey bars are 95% confidence intervals. (F) Genomic location of the TRIB SNPs that are significantly associated with sleep (red) and obesity (green). Blue corresponds to exons and grey to intronic regions.

The *Drosophila trbl* gene has three orthologues in humans (*TRIB1-3*), of which *TRIB2* is the closest orthologue (Fig. 5A). *Drosophila* and human tribbles proteins share common features, such as a unique central kinase-like domain. Human TRIB1-3 also contains a COP1 site at the carboxy terminus, which is predicted to bind COP1 E3-ubiquitin ligases. *Drosophila* Trbl diverges from human TRIB1-3 in the carboxy terminus because it lacks the COP1 binding site (reviewed by Dobens et al., 2021). Given that the three human TRIBs share common features with the single *Drosophila* Trbl, we next explored the potential associations between human *TRIB1-3* and alterations in either weight or sleep by analysing SNPs in the human TRIB genes. We used data from the UK Biobank and an *in silico* workflow to screen for associations between SNPs in *TRIB1-3* and alterations in either sleep duration or waist-to-hip ratio (WHR), a readout of obesity, accounting for covariates (Fig. 5B,C). We found several SNPs associated with differences in WHR (Fig. 5D) and sleep duration (Fig. 5E) in *TRIB1-3* after correcting for the false discovery rate (FDR) (Fig. 5F).

**Fig. 6.**
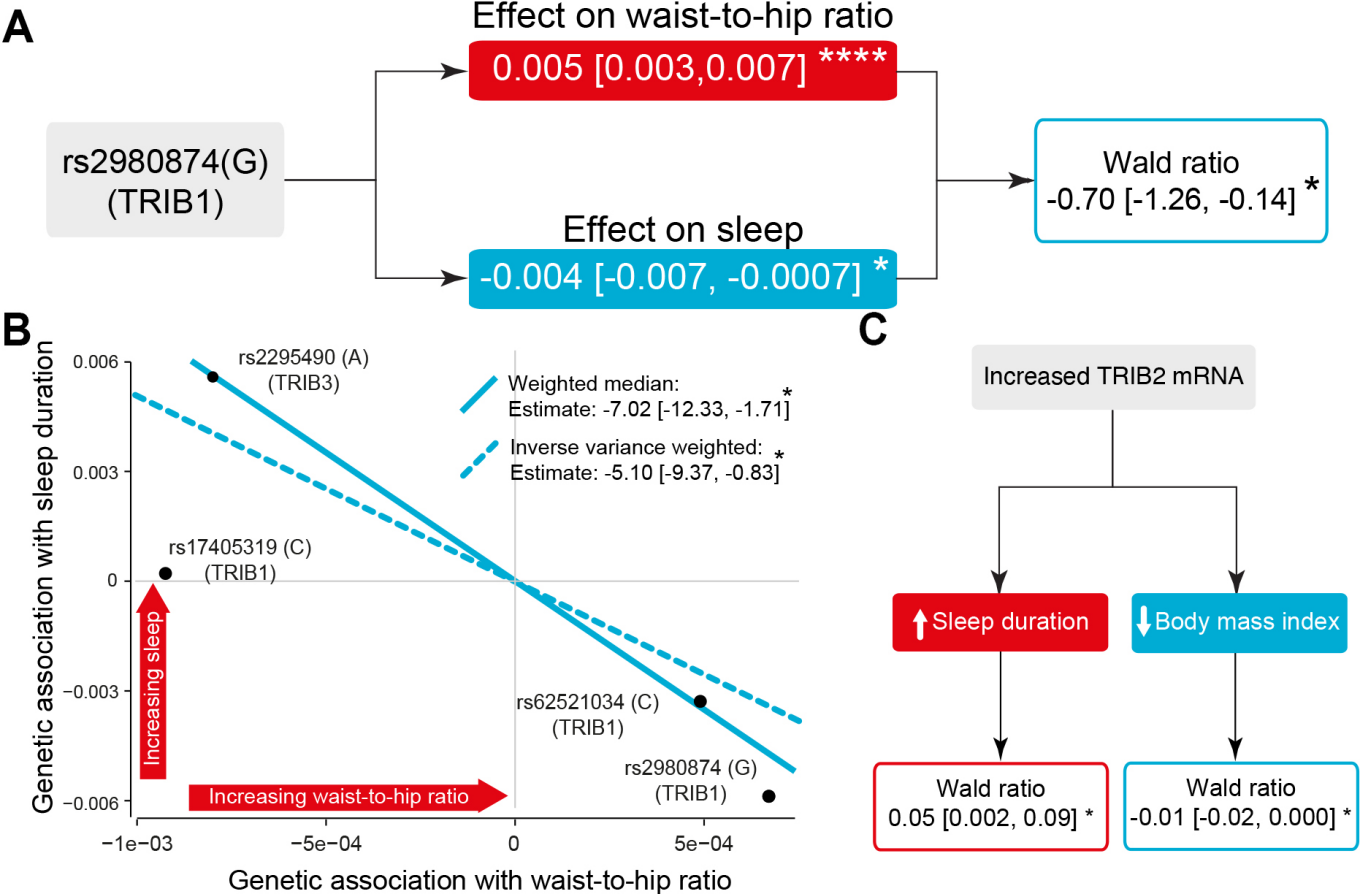
**Higher TRIB mRNA levels are linked to the inverse relationship between weight and sleep duration.** (A) The rs2980874 (G) variant in *TRIB1* is significantly associated with a higher WHR and shorter sleep duration [scaled estimate (95% confidence intervals); *P<0.05, ****P<0.0001; linear regression). The causal estimate of the effect of WHR on sleep is calculated by dividing its gene–outcome association by its gene–exposure association [estimate (95% confidence intervals); *P<0.05, Wald test]. Red and blue correspond respectively to positive and negative associations, respectively. (B) Mendelian randomisation analysis of all significant genetic instruments for WHR and their effect on sleep duration [estimate (95% confidence intervals); *P<0.05; weighted median and inverse variance weighted]. (C) Mendelian randomisation analysis of *TRIB2* variants correlated with increased mRNA levels, and their effect on weight (BMI) or sleep duration [estimate (95% confidence intervals); *P<0.05, Wald test]. The SNP instrument used for increased *TRIB2* mRNA and weight is rs17390839 (A). The SNP instrument used for increased *TRIB2* mRNA and sleep duration is rs72773697 (A).

Given these associations, we next investigated whether SNPs in TRIB genes mediate the inverse correlation between weight and sleep duration. We found that rs2980874 (G) was significantly associated with a higher WHR and a shorter sleep duration, indicating a negative association between sleep duration and WHR (Fig. 6A). We next analysed all variations significantly associated with WHR (Fig. 5D) and their relationships with sleep duration. We used weighted median and inverse variance estimators to account for biases in our methods. We found that genetic variations in *TRIB1* and *TRIB3* mediate the inverse relationship between WHR and sleep duration (Fig. 6B). These results show that genetic variations in TRIB genes associated with higher levels of obesity markers are also associated with a shorter sleep duration, supporting our observation of the inverse relationship between sleep and weight in *Drosophila*.

Next, as *Drosophila* Trbl shows the highest homology to TRIB2, we examined whether increased expression of *TRIB2* is also linked to alterations in weight and sleep in humans. We used data from the Genotype-Tissue Expression Project (GTEx) (GTEx Consortium, 2013). We combined this information with genome-wide associations with body mass index (BMI) from the GIANT consortium (Yengo et al., 2018) and data on sleep duration from UK Biobank participants (Jones et al., 2016). We found that SNPs associated with higher *TRIB2* expression were linked to both decreased BMI and increased sleep duration (Fig. 6C). We conclude that this genetic information on human *TRIB1-3* shows an inverse association between sleep duration and obesity, validating our observations in flies.

### The TRIB3 Q84R variant is linked to shorter sleep duration in humans and flies

**Fig. 7.**
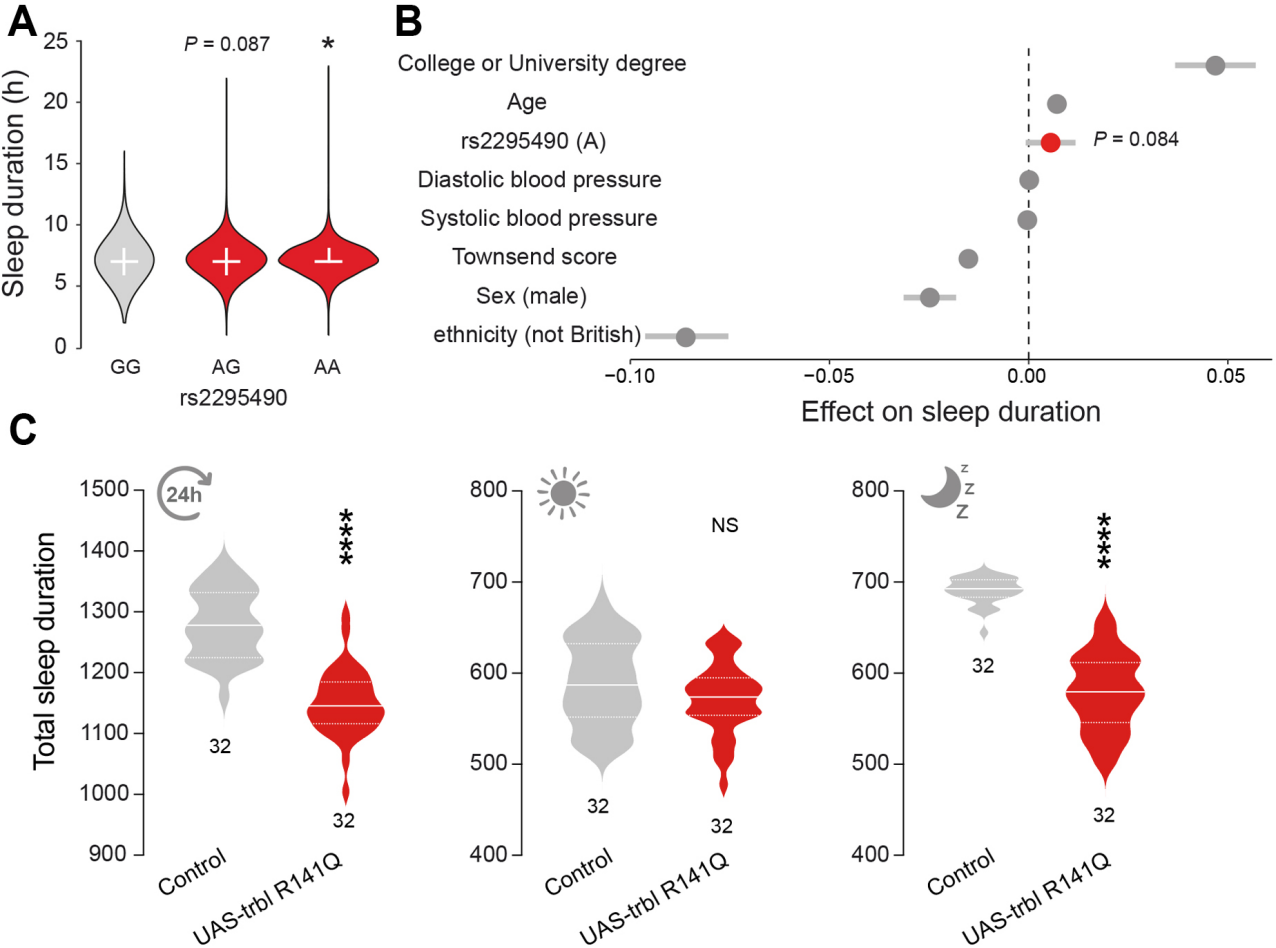
**The *TRIB3* rs2295490 genetic polymorphism is also linked to sleep duration.** (A,B) The rs2295490 (A) variant, coding for an R residue, is significantly associated with a longer sleep duration in UK Biobank participants (A; *P<0.05; unpaired *t*-test) and remains after accounting for covariates [B; scaled estimate (95% confidence intervals); linear regression]. The error bars in grey show the 95% confidence intervals of the estimated effect (circles). The investigated variable is in red. (C) Expression of the R-to-Q mutant *trbl* gene in flies is linked to less sleep during the dark phase. Effects of *trbl R141Q* expression on total sleep duration during 24 h (left; ****P<0.0001; unpaired *t*-test), during the day (middle; NS, not significant; unpaired *t*-test) and during the night (right; ****P<0.0001; Mann–Whitney test) are shown. Genotypes: *w; pplGal4/+; +* (control), *w; pplGal4/+; UAStrbl-R141Q/+* (UAS-trbl R141Q).

The rs2295490 SNP in *TRIB3* is robustly associated with T2DM (Prudente et al., 2009) and T2DM-linked complications (He et al., 2016; Zhou et al., 2019). Because we found a link between rs2295490 and WHR, we next investigated whether this SNP is also associated with alterations in sleep. The *TRIB3* (A) allele of rs2295490 encodes for Q at position 84, and the (G) allele encodes for R. We showed that rs2295490 (Q84) is linked to a lower WRH ratio (Fig. 6B; see also https://github.com/M1gus/Tribbles-sleep). We conducted an univariate analysis and found that, compared with R84, Q84 is associated with an increase in sleep duration (Fig. 6B and Fig. 7A) and this trend remained after accounting for several confounding variables (Fig. 7B).

*Drosophila trbl* was used to investigate the role of the Q84R polymorphism in *TRIB3.* R at this position (R141) is the predominant variant in flies whereas Q (Q84) is the main variant in humans (Fischer et al., 2017). Studies focusing on TRIB3 show that the Q84R variant represents a gain-of-function alteration in this protein (Andreozzi et al., 2008; Fischer et al., 2017; Prudente et al., 2009). We therefore tested the consequences of expressing the R141Q version of Trbl in the fat body of flies and found that it caused a reduction in sleep duration during the dark phase (Fig. 7C). We conclude that this polymorphism is linked to differences in sleep duration across flies and humans.

## DISCUSSION

Both human TRIB3 and the *Drosophila* orthologue Trbl have been reported to act as negative regulators of insulin signalling by directly binding to Akt kinase, a downstream effector of the InR. They block phosphorylation-dependent Akt activation, which is important for the transduction of signals from the InR (Das et al., 2014; Du et al., 2003). TRIB3 is upregulated in obese adults and in animal models of this disease as well as in insulin-resistant models (reviewed by Kiss-Toth, 2011). Additionally, TRIB3 polymorphisms are associated with T2DM and insulin resistance (Liu et al., 2012; Oberkofler et al., 2010; Prudente et al., 2009).

It was reported that Trbl can block insulin signalling by inhibiting Akt, a downstream effector of insulin signalling (Das et al., 2014). Additionally, overexpression of the Q84R polymorphic allele of *TRIB3* in mammalian cells reduces insulin exocytosis (Liew et al., 2010).

In this study, we show that the expression of *trbl* mediated by the *ppl-*Gal4 driver, a tool commonly used to drive the expression of transcripts in the fat body of flies, leads to a reduction in insulin signalling, reminiscent of phenotypes associated with insulin-resistant diabetes in mammals. We also observed that, in addition to metabolic defects, Trbl expression in the fat body leads to increased levels of *takeout*, which encodes a *Drosophila* hormone controlled by the circadian clock that is induced upon starvation. This suggests a relationship between animal nutrient state and behaviour.

The insulin signalling cascade controls several developmental processes (reviewed by Garofalo, 2002; Koyama et al., 2013). Starvation of *Drosophila* larvae before they reach critical weight, or systemic suppression of the insulin signalling pathway, or ablation of IPCs delays the onset of metamorphosis (Rulifson et al., 2002; Shingleton et al., 2005). We show that ubiquitous expression of *trbl* causes a decrease in the number of developed pupae at day 10 AEL, indicating that increasing *trbl* expression leads to a developmental delay, consistent with previous reports (Das et al., 2014). However, when Trbl overexpression is restricted to adult flies, it causes the accumulation of TAGs and insulin resistance, indicating that the metabolic defects observed herein are not due to developmental impairment (Hong et al., 2016).

We further show that overexpression of *trbl* decreases the lifespan of flies under normal dietary conditions, but increases the lifespan of flies under starvation. In mice, starvation increases *Trib3* expression (Matsushima et al., 2006). We reason that the higher levels of TAGs linked to the overexpression of Trbl can provide increased levels of fatty acids for β-oxidation, a mechanism through which cells can generate energy during periods of fasting. The decreased lifespan of flies fed a normal diet was more apparent in flies expressing Trbl using the fat body driver (*ppl*-Gal4) than in flies expressing Trbl using a ubiquitous driver (*da*-Gal4). The cause of this difference is unknown; however, it may be related to differences in the amounts of Trbl expression between the two drivers.

*Drosophila* mutants for effectors of the insulin signalling pathway, including *Akt* and *DILP* mutants, show a significant reduction in body weight (Grönke et al., 2010; Murillo-Maldonado et al., 2011). We demonstrate that fat body expression of *trbl* causes a decrease in the body weight of flies, further supporting the role of *trbl* as a negative regulator of insulin signalling.

Insulin signalling controls lipid metabolism, and we show that upregulating Trbl in the *Drosophila* fat body increases TAGs, whereas Trbl silencing lowers their levels. We also demonstrate that Trbl upregulation does not alter nutrient intake. In mammalian and *Drosophila* adipose tissue, inhibition of Akt leads to nuclear translocation of the transcription factor FOXO and transcriptional upregulation of either mammalian adipose triglyceride lipase (ATGL; PNPLA2) or fly Brummer (Bmm), the main lipase responsible for the hydrolysis of TAGs (Chakrabarti and Kandror, 2009; Grönke et al., 2005; Schweiger et al., 2006). Therefore, by inhibiting Akt, Trbl should activate FOXO and promote lipolysis rather than lipid storage, as observed in our study. However, *InR*, *chico* and *DILP2,3,5* mutants, as well as flies with ablated IPCs, show an increase in TAG levels (Böhni et al., 1999; Broughton et al., 2005; Grönke et al., 2010; Tatar et al., 2001), suggesting that a systemic decrease in insulin signalling increases rather than decreases lipid levels, as observed in our study.

Here, we demonstrate that Trbl overexpression causes a decrease in the levels of DILP2, suggesting that it also acts upstream of Akt by decreasing total insulin levels in flies. Through inter-organ communication, the fat body can control insulin release from IPCs in the brain via humoral signalling (Géminard et al., 2009). Eiger is a fat body hormone and TNFα orthologue that can inhibit DILP production by IPCs harbouring Grindelwald, its receptor (Agrawal et al., 2016). We show that Trbl overexpression causes a decrease in the systemic levels of DILP2. It is therefore possible that increased expression of Trbl mediated by the *ppl* driver might decrease the levels of Eiger and thus remotely control DILP levels in IPCs. We found that the *ppl*-Gal4 driver is likely to cause expression of Trbl in presynaptic neurons that form connections with IPCs. Therefore, we cannot rule out the possibility that the loss of DILP2 observed upon overexpression of Trbl using the *ppl*-Gal4 driver is a consequence of the expression of Trbl in this population of neurons as well as in the fat body. It would be important to determine the effect of fat body expression of Trbl on DILP2 levels using an independent fat body driver that is not confounded by secondary sites of Gal4 expression.

The *Drosophila* fat body regulates complex behaviours, including feeding (Kim et al., 2017), courtship behaviour (Lazareva et al., 2007) and sleep (Yurgel et al., 2018). Unpaired 2 (Upd2), a functional homologue of leptin, is secreted by the fat body and modulates sleep (Ertekin et al., 2020; Rajan and Perrimon, 2012). Our results show that increasing the expression of *trbl* leads to an increase in sleep and the upregulation of *takeout*, a circadian hormone that also serves as a starvation marker, whereas silencing this pseudokinase leads to less sleep. Takeout signalling occurs via a circadian output pathway that integrates information about time and nutrient status into feeding activities (Sarov-Blat et al., 2000). It is conceivable that this hormone functions in the fat body, connecting metabolic reprogramming to the alterations in activity that we observe upon upregulation of Trbl in the fat body of flies.

In our analysis of *TRIB1* using UK Biobank data, we identify three intronic SNPs (rs2980874, rs7833718 and rs6470353) and two exonic SNPs (rs62521034 and rs17405319)*. TRIB1* mRNA has a short half-life of less than 1 h, which is a feature of genes with regulatory functions (Sharova et al., 2009). Intronic DNA variants that affect mRNA levels are common (Kim et al., 2014) and can act by regulating the splicing of precursor mRNAs (ElSharawy et al., 2006). Among the variants included in our analysis, only rs2980874 (G) is significantly associated with both increased obesity and a lower sleep duration (Fig. 6A). The two exonic SNPs in *TRIB1* are both non-protein coding and located in the 3′ untranslated region, a hotspot for post-transcriptional regulation via microRNAs (Niespolo et al., 2018). This suggests that these two exonic SNPs could be involved in the microRNA-based modulation of *TRIB1* mRNA levels.

The SNPs within *TRIB2* reported in our study are all intronic, and the SNPs linked to *TRIB2* mRNA levels from the GTEx database are located outside of the *TRIB2* gene. The majority of genetic variants identified via genome-wide association studies fall within noncoding regions of the human genome (Edwards et al., 2013). This indicates that they may reside within regulatory elements of the genome, such as long noncoding RNAs (lncRNAs) or microRNAs (Edwards et al., 2013). Both of the SNPs that we found to be associated with higher *TRIB2* mRNA levels as well as sleep duration and obesity (rs72773697 and rs17390839; Fig. 5) are located outside of the *TRIB2* gene locus. rs72773697 is located 11,376,426 bases downstream of the *TRIB2* open reading frame and maps to an uncharacterised lncRNA (LOC105373438). It is possible that LOC105373438 modulates the expression of TRIB2 and that rs72773697 could therefore act by altering the ability of LOC105373438 to modulate TRIB2 expression. rs17390839 is located 17,919 bases downstream of the *TRIB2* open reading frame in a noncoding region and could alter *TRIB2* transcription.

The SNPs in *TRIB3* reported in our study consist of two intronic (rs6076472 and rs6051597) and two exonic (rs2295490 and rs11537605) SNPs. One of the SNPs in *TRIB3* (rs2295490), associated with an increase in sleep and decrease in WHR according to our data, is a previously characterised polymorphism, Q84R. This polymorphism is associated with a 32% increase in the probability of developing early-onset T2DM (Prudente et al., 2009) and decreased insulin levels (Liew et al., 2010). Insufficient insulin levels can prevent glucose metabolism and decrease the availability of energy. When this occurs, the body can use stored fat for energy production, causing a reduction in body weight (reviewed by Rosen and Spiegelman, 2006), in line with our results in flies overexpressing *trbl*. We further analysed the sleep patterns of flies overexpressing *trbl* with either Q or R at position 141 to model the role of the Q84R polymorphism in *TRIB3*. We found that the Q variant is linked to less sleep whereas the R variant caused an increased sleep duration, both specifically during the dark phase. These observations support our model using human medical data, and highlight a phenotypic link between TRIB polymorphisms and sleep duration. Further work could explore whether *trbl* alters sleep patterns by modulating insulin levels and the activity of sleep-regulating neurons via a potential communication route between the fat body and sleep-regulating neurons.

We show that the rs11537605 (G) SNP in *TRIB3* is significantly associated with a longer sleep duration. This synonymous variant leads to a change in the leucine codon from CTG to CTA. Previous research demonstrated that there is a codon bias towards CTG in both mice and humans and that replacing CTA with CTG increases protein synthesis by 20-fold (Newman et al., 2016). It is therefore possible that the rs11537605 (G) SNP in *TRIB3* acts by altering the rate of protein synthesis from its transcript.

Our data, obtained by combining both *in vivo* and *in silico* approaches, indicate that Tribbles proteins play a role in the regulation of body weight and sleep, both of which are markers of T2DM. We suggest that higher Trbl expression levels could confer protection against stress conditions, such as starvation, but lead to metabolic dysfunction under non-stress conditions. Further research is required to determine the mechanisms by which SNPs identified in our study could modify TRIB mRNA levels.

## MATERIALS AND METHODS

### Genetics and *Drosophila* strains

Fly stocks and crosses (unless otherwise stated) were maintained on standard cornmeal agar media at 25°C. The following strains were used: *w; +; daGal4*, *w; gmrGal4; +*, *w; pplGal4; +*, *yw, UAS-myrGFP* and *QUAS-mtdTomato-3xHA; trans-Tango; +* (all from Bloomington *Drosophila* Stock Center); *w; UASTrbl-RNAi; +* (Vienna *Drosophila* RNAi Center; VDRC_*P{KK108667}VIE-260B*); *w; +; UAS-trbl R141Q* (gift from L. Dobens, University of Missouri-Kansas City, Kansas City, MO, USA); and *yw; +; ILP2-HF* (gift from A. Telemans, DKFZ, Heidelberg, Germany). Haemagglutinin (HA)-tagged cDNA fragments encoding full-length *trbl* (ID: RH69304) from the *Drosophila* Genomics Resource Center were cloned into the pUASTattB vector for PhiC31-mediated site-directed transgenesis. Transgenic flies were generated at the Cambridge Fly Facility, Department of Genetics, University of Cambridge, UK. All the experiments on adult flies were performed using males.

### Lifespan analysis

Groups of 12 newly eclosed males of each genotype were placed into separate vials with food and maintained at 25°C. The flies were transferred to vials containing fresh food every 2-3 days, and the number of dead flies was recorded. The data are presented as Kaplan‒Meier survival distributions, and significance was determined by log-rank tests.

### Climbing assay

Climbing assays were performed using a counter-current apparatus equipped with six chambers. A total of 10-15 male flies were placed into the first chamber, tapped to the bottom, and then allowed 20 s to climb a distance of 10 cm. The flies that successfully climbed 10 cm or beyond within 20 s were then transferred to a new chamber, and both sets of flies were given another opportunity to climb the 10-cm distance. This procedure was repeated a total of five times. After five trials, the number of flies in each chamber was counted to calculate the climbing index. A video demonstrating this technique can be found at https://youtu.be/vmR6s_WAXgc. The climbing index was measured using a weighted average approach with the following formula:
(1)

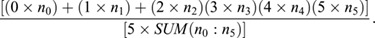
In this formula, *n*_0_ corresponds to the number of flies that failed the first trial, and *n*_1_-*n*_5_ are the numbers of flies that successfully passed each successive trial. At least 100 flies were used for each genotype tested.

### CAFE assay

Eight male flies were placed into an experimental vial (8 cm height, 3.3 cm diameter) containing six microcapillary tubes (BRAND^®^ disposable BLAUBRAND^®^ micropipettes, intraMark, BR708707, with 1 µl marks) containing 5 µl of liquid food. The experimental vials were placed in a plastic box with a cover to control humidity. Liquid food was prepared by dissolving 50 mg of yeast granules in 1 ml of boiling water by vortexing, followed by brief centrifugation (14,000 ***g*** for 1 min). Then, 40 mg of sucrose (Sigma-Aldrich, 84097) was added to 800 µl of the dissolved yeast mixture, followed by vortexing. The microcapillary tubes were filled with liquid food up to the 5 µl mark. Each experiment consisted of five experimental vials per genotype. The flies were acclimatised in the experimental vial without any food for 2 h prior to the start of the experiment. This step was also used to incentivise the flies to eat once the food was introduced. The flies were allowed to feed for 19 h, after which the amount consumed (in mm) was measured with a digital calliper (Dasqua Bluetooth Digital Calliper 12″/300 mm, 24108120). The total amount of food consumed was calculated using the formula:
(2)

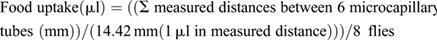


### Locomotor assays

Ten-day-old males were individually loaded into *Drosophila* Activity Monitors (DAM5) within 8×65-mm2 glass Pyrex tubes (TriKinetics) containing normal fly food. The flies were maintained at 25°C under a 12-h/12-h light/dark cycle for at least 7 days. Sleep and activity data were collected every 30 s and analysed using the Sleep and Circadian Analysis MATLAB Program (SCAMP) developed by the Griffith laboratory (Donelson et al., 2012). Analyses were performed for 7 days starting at the first Zeitgeber time (ZT0) to allow acclimation. Dead flies and flies with rhythmic index scores <1 were removed from the analyses.

### Starvation resistance assay

The assay was performed using TriKinetics. Sixteen male flies (14 days old) were placed into tubes containing 2% agar. Activity was monitored until death using the DAM system (TriKinetics). Sleep and activity data were collected every 30 s (bout size). The time of death was manually determined for each individual fly as the last bout of waking activity.

### Counting the number of pupae

To assess the effect of transgene overexpression on fly development, the number of developed pupae at day 10 AEL was counted. Following mating at 25°C, the parental flies (ten virgin females and three males) were kept for 2 days and were flipped into vials with fresh food every 2 days for all genotypes. Vials were kept for 10 days to allow fertilised eggs to develop into mature pupae. The number of pupae per vial was counted at day 10 AEL for all genotypes.

### Measurements of fly body weights

To measure fly body weights, seven adult males and seven adult females were anesthetised and weighed separately in a plastic tray. The weights were obtained at four different time points (3, 7, 14 and 25 days old). At least three experiments per genotype were performed. The body weight, in mg, was normalised to the number of flies measured.

### RNA extraction and quantitative real-time PCR

Flies were snap-frozen in liquid nitrogen at ZT4 and stored at −80°C until RNA extraction. RNA was extracted using the phenol–chloroform extraction method. Five to six male flies were transferred to an RNase-free tube (Ambion, AM12400) with 100 µl of TRIzol (Ambion, 15596018) and snap-frozen in liquid nitrogen. Samples were homogenised with a motor pestle for 2 min, after which an additional 500 µl of TRIzol was added. Then, 0.2 volumes of chloroform (Sigma-Aldrich, C2432) were added to the samples, followed by vortexing for 15 s, incubation for 3 min at room temperature (RT), and centrifugation for 15 min at 17 ***g*** at 4°C. The upper phase was transferred to a fresh tube to which 0.7 volumes of isopropanol (Sigma-Aldrich, 59304) were added, and the samples were left overnight at −20°C to precipitate the RNA. Thereafter, the samples were centrifuged at 17 ***g*** for 10 min at 4°C and washed twice with 600 µl of 70% ethanol (Sigma-Aldrich, 32221-M) in nuclease-free water (Ambion, AM9906). Following 5 min of centrifugation at 7.8 ***g*** at 4°C, the pellet was air-dried for approximately 15 min and resuspended in 30 µl of preheated (55°C) nuclease-free water. The RNA concentration was quantified with a Nanodrop instrument (NanoDrop™ 2000/2000c Spectrophotometers, Thermo Fisher Scientific, ND-2000).

Quantitative real-time PCR with reverse transcription (qRT-PCR) was performed on a real-time cycler (7500 Fast Real-Time PCR Systems, Applied Biosystems, 4351106) using the SensiFAST SYBR Lo-ROX One-Step Kit (Bioline, BIO-74005). Two microlitres of 25 ng/µl RNA from each sample was loaded onto a qPCR plate in triplicate. Then, 18 µl of master mix (comprising 1.6 µl of forward and reverse gene-specific primer mix, 5.8 µl of DEPC-treated water, 10 µl SensiFAST SYBR^®^ One-Step mix, 0.2 µl of reverse transcriptase and 0.4 µl of RiboSafe RNase Inhibitor) was added to each well. Fold change values were calculated using the comparative Ct method (Schmittgen and Livak, 2008). For RT-qPCR, we measured the coefficient of variation (CV) of the technical replicates and excluded any samples with CV >3% from statistical analysis. The following *Drosophila* gene-specific primers were obtained from QIAGEN: Dm_CG6283_1_SG QuantiTect Primer Assay (QT00983689), Dm_InR_2_SG QuantiTect Primer Assay (QT00979384), Thor RT² qPCR Primer Assay for Fruit Fly (NM_057947) (PPD02171A-200), Dm_to_1_SG QuantiTect Primer Assay (QT00982415), trbl RT2 qPCR Primer Assay for Fruit Fly (PPD10848A). Gene-specific primers for the housekeeping gene *rp49* (*RpL32*) (forward, 5′-TGTCCTTCCAGCTTCAAGATGACCATC-3′; reverse, 5′-CTTGGGCTTGCGCCATTTGTG-3′) were ordered from Sigma-Aldrich.

### Immunofluorescence analysis

For the detection of Trbl-HA, the bodies of larvae were inverted to expose internal tissues to the buffer and were fixed in 4% paraformaldehyde (Electron Microscopy Sciences, 15710) in PBS for 20 min. The tissue was then washed three times for 10 min each in PBT [PBS with 0.2% Triton X-100 (BDH Laboratory Supplies, 306324N)], followed by blocking at 4°C overnight in PBT with 10% normal goat serum (NGS) (Life Technologies Corporation, PCN5000). Primary antibody [rabbit anti-HA (Sigma-Aldrich, H6908)] incubation was carried out at 1:100, and secondary antibody [Alexa Fluor™ 488 F(ab′)2 fragment of goat anti-rabbit IgG (H+L) (Invitrogen, A11070)] at 1:200 dilution, both in PBT with 10% NGS at 4°C overnight. After four 10-min washes, the samples were kept in PBT at 4°C until mounting. Eye discs were dissected and mounted on coverslips in Vectashield (Vector Laboratories, H-1000).

For DILP2 and *trans*-Tango staining, whole flies were fixed in 4% paraformaldehyde and 1% Triton X-100 with rotation overnight at 4°C. The flies were washed three times for 10 min each wash in 0.1% Triton X-100/PBS, after which the brains were dissected in chilled PBS and washed again three times for 15 min per wash in PBS/1% Triton X-100. The brains were incubated in a blocking solution (10% NGS/0.5% Triton X-100/PBS) at 4°C overnight. Incubation with the primary antibodies [DILP: anti-FLAG (1:500), Sigma-Aldrich, F3165; *trans*-Tango: chicken anti-GFP (1:1000), Abcam, AB13970, and anti-HA rat (Roche, 11867423001; 1:100), Sigma-Aldrich, H6908] was then conducted in the blocking solution overnight. Following three 15-min washes in 0.5% Triton X-100/PBS, brains were incubated in secondary antibodies [Alexa Fluor™ 488 goat anti-mouse IgG (H+L) (Invitrogen, A11029), at 1:250; *trans-*Tango: Alexa Fluor 488 goat anti-chicken, Abcam, Ab150173 (1:500); goat anti-rat Alexa Fluor 647, Abcam, Ab150167 (1:500)] for 2 h at RT. After four 10-min washes, the brains were mounted on coverslips in Vectashield (Vector Laboratories, H-1000). Hoechst 33342 (1:500, Invitrogen, H3570) was used as a nuclear stain for the Trbl and DILP experiments.

### Image acquisition and processing

Images were acquired with a Zeiss LSM880 confocal microscope as uncompressed bitmapped digital data (Tiff format) and processed using Adobe Photoshop, employing established scientific imaging workflows (Sedgewick, 2008).

### Metabolic assays

The assessment of DILP2 was performed by ELISA, and levels of TAGs were assessed through colorimetric assays using 96-well microtiter plates and an Infinite M200Pro multifunction reader (Tecan).

TAG assays were essentially performed as previously described (Tennessen et al., 2014). Briefly, five adult flies were homogenised in 110 µl of 0.05% Tween 20 in PBS (PBST) for 2 min on ice and immediately incubated at 70°C for 10 min to inactivate endogenous enzymatic activity. A 35 µl fly homogenate sample and a glycerol standard (Sigma-Aldrich, G7793) were incubated together with either 35 µl of PBST (for free glycerol measurements) or 35 µl of TAG reagent (Sigma-Aldrich, T2449, for TAG measurements) at 37°C for 60 min. After 3 min of centrifugation at 17 ***g***, 30 µl of each sample was transferred into a clear-bottom plate (two technical replicates per biological sample) together with 100 µl of free glycerol reagent (Sigma-Aldrich, F6428) and incubated at 37°C for 5 min.

The DILP2 ELISA was performed as previously described (Fedele et al., 2022). The wells of a 96-well plate (Thermo Fisher Scientific, 46867) were coated with 100 µl of anti-FLAG antibody (Sigma-Aldrich, F1804) diluted in 0.2 M sodium carbonate/bicarbonate buffer (pH 9.4) to a final concentration of 2.5 µg ml^–1^ and incubated overnight at 4°C. The plate was washed twice with PBS containing 0.2% Tween 20 (Sigma-Aldrich, P1379) for 5 min at RT, after which the plate was coated with 350 µl of PBS containing 2% bovine serum albumin (Sigma-Aldrich, A3299) overnight at 4°C. The plate was washed three times with PBS containing 0.2% Tween 20 for 5 min at RT. Single-fly extracts were prepared by lysing individual flies placed in a 1.5-ml Eppendorf tube containing 100 µl of PBS with 1% Triton X-100 (BDH Laboratory Supplies, 306324N), after which the samples were left at RT for 30 min on a rotary shaker. Following centrifugation at 21,000 ***g*** at RT, 50 µl of the lysate was added to the precoated plate for overnight incubation at 4°C with gentle agitation, followed by the addition of 5 µl of anti-HA-HRP antibody (Roche Applied, 12013819001) diluted 1:350 in PBS to the lysate and brief vortexing and centrifugation (14,000 ***g*** for 1 min). The rest of the lysate was used for the Bradford assay. Samples were aspirated, and the wells were washed six times with PBS containing 0.2% Tween 20. Then, 100 µl of TMB ELISA substrate (Thermo Fisher Scientific, 34029) was added to each well and incubated on a rotary shaker for 30 min in the dark at RT. To stop the reaction, 100 µl of 2 M sulfuric acid (Sigma-Aldrich, 339741) was added, and the absorbance was measured at 450 nm on a plate reader. Subsequently, TAG and DILP2 absorbances were divided by the protein concentration of the respective sample, which was measured by Bradford assay (Sigma-Aldrich, B6916).

### Phylogenetic analysis

The analysis of evolutionary relationships between *Drosophila* and *Homo sapiens* Tribbles pseudokinases was performed using MacVector. The accession codes referring to each individual protein were sp|Q9V3Z1|TRIB_DROME, sp|Q96RU8|TRIB1_HUMAN, sp|Q92519|TRIB2_HUMAN and sp|Q96RU7|TRIB3_HUMAN. The settings for the analysis were ‘best tree’ tree settings, tie breaking set to ‘systematic’, uncorrected ‘p’ and gaps distributed proportionally. The tree is unrooted.

### Statistical analyses

Statistical analyses were performed using GraphPad Prism (www.graphpad.com). The data are presented as mean values, and the error bars indicate s.d. The number of biological replicates per experimental variable (*n*) is indicated in either the respective figure or figure legend. In violin plots, the solid line represents the median and dashed lines represent the quartiles. No sample was excluded from the analysis unless otherwise stated. Operators performing measurements were aware of groupings. Normality was assessed before deciding on which parametric or non-parametric test to use for inferential statistics. Significance is indicated as *P<0.05, **P<0.01, ***P<0.001, ****P<0.0001 and ‘NS’ for *P*≥0.05.

### UK Biobank data sources

The UK Biobank (Bycroft et al., 2018) comprises health data from over 500,000 community volunteers based in England, Scotland and Wales. Informed consent was obtained from all subjects. Briefly, between 2006 and 2010, adults aged between 40 and 69 years within proximity to one of the 22 UK Biobank recruitment centres were invited to participate. Extensive demographic, lifestyle, clinical and radiological information was collected from the included individuals. Baseline assessments also included a comprehensive series of questionnaires, face-to-face interviews, physical examinations and blood sampling, with links to electronic medical records. The full protocol is publicly available, and summary data can be viewed on the UK Biobank website (www.ukbiobank.ac.uk).

UK Biobank ethical approval was granted from the Northwest Multi-Centre Research Ethics Committee. The current analysis was approved under UK Biobank application #60124.

### Analysis of data from the UK Biobank and the GIANT consortium

To determine whether any SNPs in *TRIB1-3* identified in our cohort influenced sleep duration or WHR (analyses performed for Figs 4 and 7B), we used previously described workflows (Yu et al., 2021). Briefly, we built individual linear regressions iteratively for each *TRIB1-3* SNP, where the response variable was sleep duration or WHR, accounting for the covariates listed in Fig. 4C. The FDR was adjusted using the Benjamini‒Hochberg method (Benjamini and Hochberg, 1995). For each model, we calculated the odds ratio (OR) or risk ratio and the corresponding 95% confidence intervals to quantify the effects of the independent variables on the response variables. The analysis source code, detailed quality checks and all supplementary material are available in GitHub (https://github.com/M1gus/Tribbles-sleep).

To determine the relationship between TRIB1-3, sleep duration and WHR (analyses performed for Fig. 5), we first analysed the effect of the SNP rs2980874(G) in TRIB1 on sleep duration and WHR using linear regression. We used causal inference based on genetic polymorphism. There are three general assumptions made in this method: the genetic variant (rs2980874) is associated with the exposure (WHR); the genetic variant (rs2980874) is independent of the outcome (sleep duration) given the exposure (WHR) and all confounders of the exposure-outcome association; and the genetic variant (rs2980874) is independent of factors that confound the exposure–outcome relationship. We calculated the effect of WHR on sleep by dividing its gene–outcome association by its gene–exposure association using the Wald test (Wald, 1940). We then assessed the effect of WHR on sleep duration using all SNPs significantly associated with WHR using both a weighted median model and an inverse variance weighted model, following previously established methods (Burgess et al., 2019). To assess further the robustness of our results, we used two-sample Mendelian randomisation to investigate the effect of increased *TRIB2* gene expression on sleep duration and BMI. We selected SNP variants associated with increased *TRIB2* mRNA levels in any tissue from the GTEx database (Aguet et al., 2020) and used the Wald test to assess whether they were associated with sleep duration or BMI. The genome-wide associations of BMI came from the GIANT consortium (Yengo et al., 2018) and those of sleep duration from the UK Biobank (Jones et al., 2016).
